# Emergence of a non-sporulating secondary phenotype in *Clostridium (Clostridioides) difficile* ribotype 078 isolated from humans and animals

**DOI:** 10.1038/s41598-019-50285-y

**Published:** 2019-09-23

**Authors:** M. C. Connor, J. W. McGrath, G. McMullan, N. Marks, M. Guelbenzu, D. J. Fairley

**Affiliations:** 1 School of Biological Sciences and the Institute for Global Food Security, Queen’s University Belfast, Belfast, UK; 2 Veterinary Science Division, Agri-Food Biosciences Institute, Belfast, UK; 3 Present Address: Animal Health Ireland, Carrick on Shannon, Republic of Ireland; 4 Department of Microbiology, Belfast Health & Social Care Trust, Belfast, UK

**Keywords:** Bacteriology, Pathogens

## Abstract

*Clostridium (Clostridioides) difficile* is a Gram positive, spore forming anaerobic bacterium that is a leading cause of antibiotic associated diarrhoea in the developed world. *C*. *difficile* is a genetically diverse species that can be divided into 8 phylogenetically distinct clades with clade 5 found to be genetically distant from all others. Isolates with the PCR ribotype 078 belong to clade 5, and are often associated with *C*. *difficile* infection in both humans and animals. Colonisation of animals and humans by ribotype 078 raises questions about possible zoonotic transmission, and also the diversity of reservoirs for ribotype 078 strains within the environment. One of the key factors which enables *C*. *difficile* to be a successful, highly transmissible pathogen is its ability to produce oxygen resistant spores capable of surviving harsh conditions. Here we describe the existence of a non-sporulating variant of *C*. *difficile* ribotype 078 harbouring mutations leading to premature stop codons within the master regulator, Spo0A. As sporulation is imperative to the successful transmission of *C*. *difficile* this study was undertaken to investigate phenotypic characteristics of this asporogenous phenotype with regards to growth rate, antibiotic susceptibility, toxin production and biofilm formation.

## Introduction

*Clostridium (Clostridioides) difficile* is a Gram positive, spore forming anaerobic bacterium that is a leading cause of gastroenteritis-associated deaths and currently the most common cause of hospital acquired infection in many developed countries^1,2^. It is also a significant animal pathogen and has been identified as a cause of fatal diarrhoea and enteric disease in neonatal piglets^3,4^. *C*. *difficile* is a genetically diverse species that can be divided into 8 phylogenetically distinct clades: 1 to 5 and C-I to C-III^5,6^. Clades 1–5 contain toxigenic ribotypes capable of causing human disease^5,7^, whereas clades C-I and C-II consist only of non-toxigenic strains along with clade C-III, which is made up of mostly non-toxigenic isolates with only one toxigenic isolate recently recorded^6^.

*C*. *difficile* clade 5 is genetically distant from all other *C*. *difficile* clades, and believed to have diverged from clades 1–4 over a period of approximately 85 million years^8^. Significant variation has been noted within the Pathogenicity Locus (PaLoc) of clade 5 itself, whereby two lineages within the clade appear to have acquired their PaLoc genes independently^7^. Isolates with PCR ribotype 078 (R078) belong to clade 5, and are often associated with *C*. *difficile* in both humans and animals^9–12^. Notably, R078 is one of the most common ribotypes found causing human infection in Northern Ireland^13^. Prevalence of R078 in both animals and humans raises questions about colonisation of both hosts by this ribotype as a possible zoonosis, about modes and routes of transmission, and about reservoirs of R078 strains.

One of the key traits which enables *C*. *difficile* to be a successful pathogen is its ability to produce robust spores, capable of surviving harsh conditions and allowing the oxygen sensitive bacteria to remain viable outside the host^14^. It is known that spore-forming pathogens are highly transmissible^15^. It has emerged that 15–35% of cases of *C*. *difficile* infection (CDI) are recurring, which is partially due to these robust spores remaining viable within the gut post antibiotic treatment^16–18^. The mechanism of sporulation is similar in both *Clostridiales* and *Bacillales*, with the same key components involved; the sporulation regulator, Spo0A, and the sporulation-specific sigma factors sigA (σ^A^), sigH (σ^H^), sigF (σ^F^), sigE (σ^E^), sigG (σ^G^), and sigK (σ^K^)^15,19^. Decisions on the formation of either spores or biofilms relies mainly on the intracellular concentration of phosphorylated Spo0A, with high levels leading to spore formation and intermediate levels inducing biofilm formation (38). It is as yet unclear exactly what triggers sporulation in *C*. *difficile*. Environmental conditions such as nutrient starvation, pH and oxygen exposure may have a role to play^20^, whilst other mechanisms such as quorum sensing could also be involved^21^.

Although sporulation is imperative to transmission of oxygen sensitive bacteria, non-sporulating variants have been observed in normally sporulating bacterial species. *Bacillus anthracis* for example has been found to produce a non-sporulating variant *in vitro*, due to nutrient abundance reducing the selective pressure to produce spores^22^. Here we describe the existence of a non-sporulating population of *C*. *difficile* R078. We show that after 7 days anaerobic incubation at 37 °C R078 isolates appeared to produce a secondary colony phenotype. These secondary colonies have a smooth, grey appearance, with a much larger, spreading colony morphology whilst microscopic examination suggested this secondary phenotype may be non-sporulating. As sporulation is imperative to the successful host-to-host transmission of *C*. *difficile* this study was undertaken to investigate counter-intuitive observation.

## Results

### Emergence of secondary phenotype

**Figure 1:**
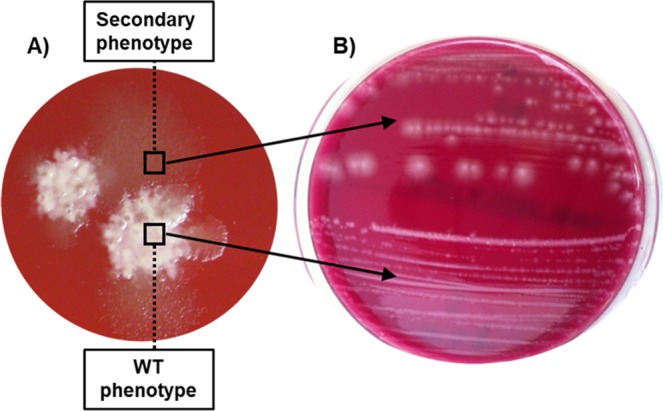
(**A**) Non sporulating variant of *C*. *difficile* 078 emerging from the wild type culture after 7 days incubation at 37 °C under anaerobic conditions on FAABL. (**B**) Both colony variants can be sub-cultured independently after initial separation (24 h incubation at 37 °C under anaerobic conditions on FAABL). Wild type cells give rise a small, rough, white colony type whist the non-sporulating variants produce much larger, smooth, grey colonies.

**Figure 2:**
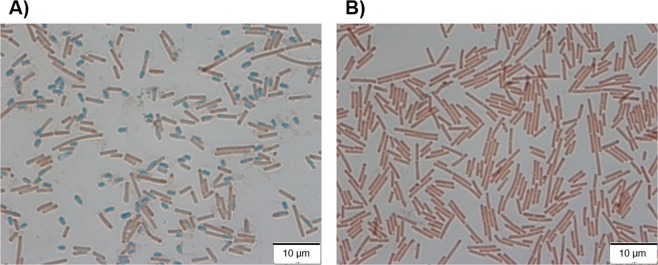
(**A**) Spore-stain image of H^s^ isolate at x 1000 magnification. (**B)** Spore-stain image of H^ns^ (non-sporulating) secondary phenotype at x 1000 magnification. Spores are identified by observation of green staining, while vegetative cells are red/pink.

R078 isolates produced a morphologically different, secondary colony variant after incubation for 7 days on fastidious anaerobe agar with horse blood (FAABL). Colonies of the wild-type phenotype (A^s^; H^s^) were observed after 48 h growth on FAABL under anaerobic conditions (Fig. 1). Further investigation of these colonies using microscopy showed the presence of spores as well as vegetative cells (Fig. 2A). In contrast, the secondary colony variants (A^ns^; H^ns^), which emerged after 7 days anaerobic incubation at 37 °C on FAABL (Fig. 1), showed the presence of vegetative cells only (Fig. 2B). Both phenotypes display a distinct colony morphology, with the (initial) sporulating phenotype giving rise to a small, rough, white colony and the non-sporulating variant a much larger, smooth, grey colony (Fig. 1B). The non-sporulating variant appears to arise as sub-populations or micro colonies within the sporulating colonies and can be sub-cultured independently. No reversion to a sporulating phenotype was observed after at least five serial subcultures of the non-sporulating variants. Of ten R078 isolates tested for the appearance of the secondary phenotype, all ten isolates showed the emergence of the secondary phenotype. To further investigate the secondary R078 phenotype, one human R078 isolate and one animal R078 isolate were selected for further detailed study.

### Multi-locus variable number tandem repeat analysis

To rule out the possibility that the secondary phenotype was due to contamination or a mixed culture, MLVA was performed on each isolate obtained. MLVA confirmed the R078 strains used were distinct from each other, however, the phenotypic variants could not be distinguished using MLVA. The sporulating (A^s^; H^s^) and non-sporulating phenotypes (A^ns^; H^ns^) gave identical MLVA profiles for both pairs of human and animal isolates (Table 1). This is the expected result if the non-sporulating isolates were mutants derived from the same parental strains in each case.

**Table 1:** MLVA profiles of animal and human isolates; A^s^, A^ns^, H^s^ and H^ns^.

	MLVA profile						
**Isolate**	**A6** ^**a**^	**B7**	**C6**	**E7**	**F3**	**G8**	**H9**
A^s^	—	21	31	5	5	10	2
A^ns^	—	21	31	5	5	10	2
H^s^	—	17	9	9	5	7	2
H^ns^	—	17	9	9	5	7	2

^a^The A6 allele is consistently absent from R078 isolates (39).

### Assessing sporulation in A^s^, A^ns^, H^s^ and H^ns^

**Figure 3:**
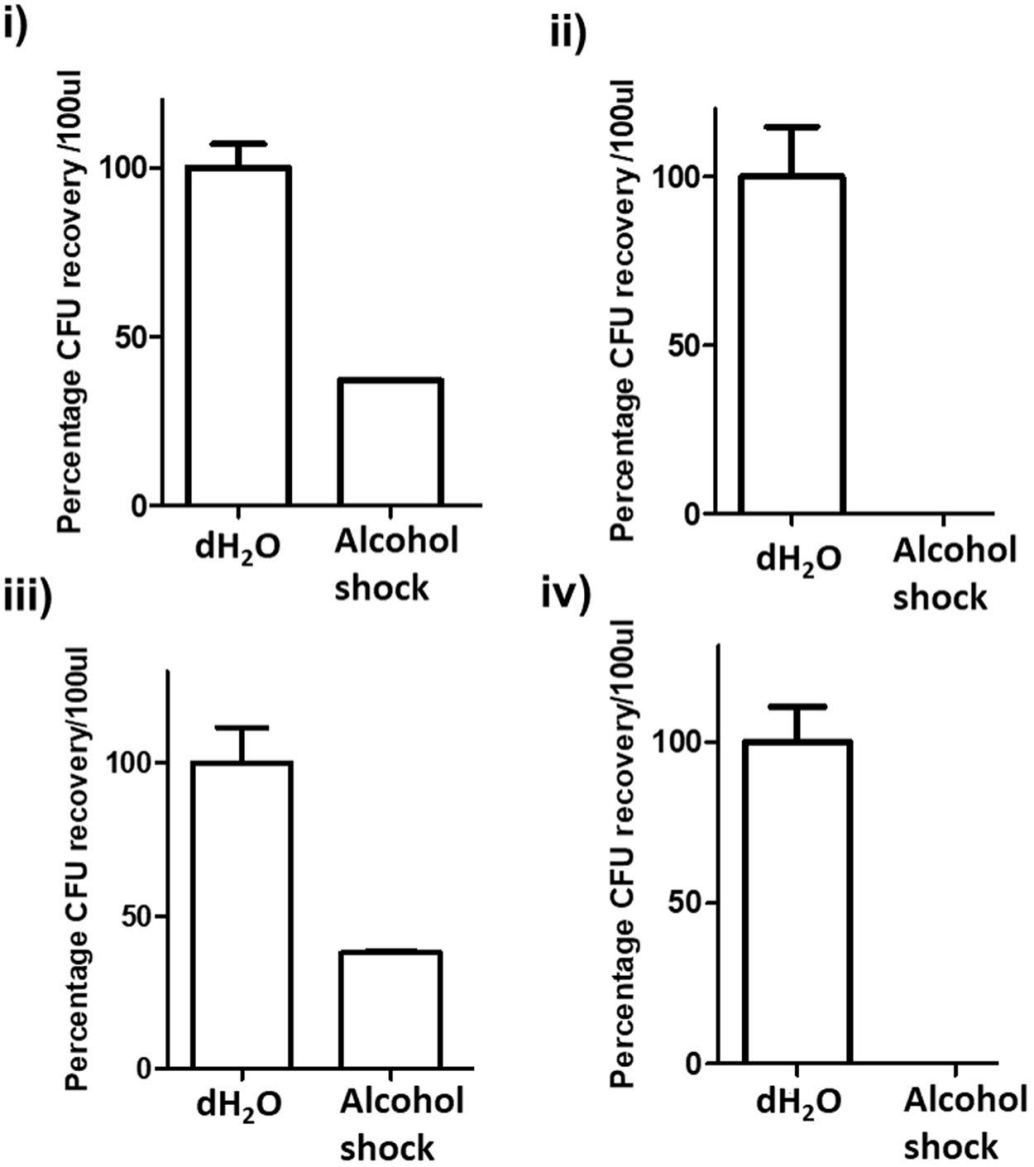
Recovery of spores after alcohol shock with 70% (v/v) ethanol for 1 h at room temperature compared to the control, CFU obtained without alcohol shock, **(C**). (**i**) A^s^ isolate, (**ii)** A^ns^ isolate, **(iii)** H^s^ isolate, **(iv)** H^ns^ isolate.

Alcohol shock survival was used to investigate sporulation within the parental strains and proposed non-sporulating variants. Relative to water-only (no alcohol) controls, alcohol shock significantly reduced the number of CFU recovered for all isolates; A^s^ (*p* < 0.01) and A^ns^ (*p* < 0.001) and H^s^ (*p* < 0.01) and H^ns^ (*p* < 0.001). An average of 37.5% ± 0.5% CFU were recovered from A^s^ and H^s^ after alcohol shock (Fig. 3[i,iii]), indicating the presence of spores within the parental strains. Viable counts from sporulating variants after alcohol shock were dramatically reduced compared to controls due to killing of vegetative cells in the cultures (Fig. 3[i,iii]). After alcohol shock of the A^ns^ and H^ns^ variants, viable counts were zero, indicating complete absence of spores (Fig. 3[ii,iv]).

### Growth rate analysis of A^s^, A^ns^, H^s^ and H^ns^

**Figure 4:**
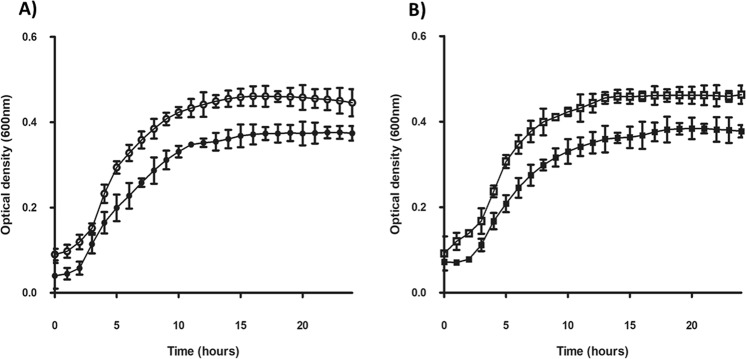
Growth of A^s^, A^ns^, H^s^ and H^ns^ in BHI over 24 h at 37 °C under anaerobic conditions. **A)** A^s^ (o) and A^ns^ (●), **B)** H^s^ (**□**) and H^ns^ (■).

The growth rate of each phenotype was investigated (Fig. 4) with no significant difference noted between A^s^ and A^ns^ (*p* > 0.05) and H^s^ and H^ns^ (*p* > 0.05). Growth rates obtained for each isolate were as follows; A^s^: 0.24 ± 0.02 h^−1^, A^ns^: 0.28 ± 0.04 h^−1^, H^s^: 0.20 ± 0.02 h^−1^, H^ns^: 0.25 ± 0.02 h^−1^. The sporulating isolates gave higher optical density readings, likely due to the presences of spores.

### Crystal violet biofilm assay

**Figure 5:**
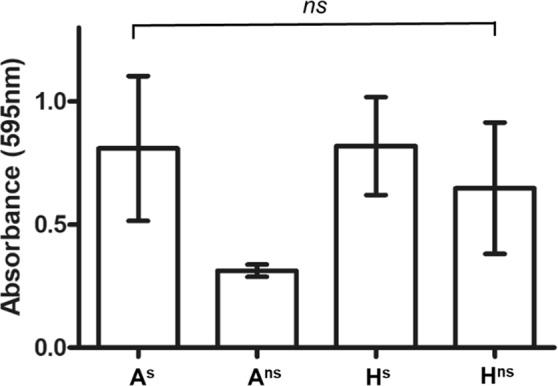
Biofilm formation of each isolate measured via Crystal Violet assay^23^. Cultures were grown statically for 6 days in BHI under anaerobic conditions at 37 °C.

To assess if any significant difference in biofilm production was associated with loss of the sporulation phenotype a Crystal Violet assay was employed^23^, to allow for the quantification of biofilm production by A^s^ and A^ns^, and H^s^ and H^ns^. If linked, a sub-population displaying increased biofilm formation could confer a plausible population-level advantage on strains exhibiting a non-sporulating behaviour. However, although variation was observed in biofilm production, it was not sufficient to conclude a significant difference in biofilm production (Fig. 5).

### Toxin production in A^s^, A^ns^, H^s^ and H^ns^

**Figure 6:**
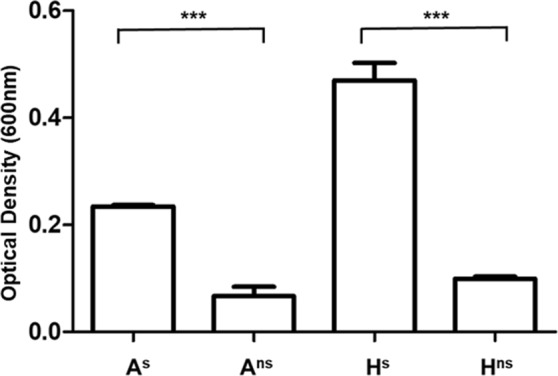
Maximum toxin production of each phenotype for animal and human isolates. Isolates were grown in BHI media under anaerobic conditions at 37 °C. Sampling began at 14.5 h after inoculation with samples taken every 3 h thereafter, for a total of 24 h: Maximum toxin production was observed at 14.5 h.

The ability of each isolate (A^s^, A^ns^, H^s^ and H^ns^) to produce toxin was assessed to determine any difference in toxigenicity between the variants. Whilst all isolates produced toxin, with a maximum level measured at 14.5 h (indicating the beginning of stationary phase); sporulating isolates (A^s^; H^s^) produced significantly more toxin than the non-sporulating isolates (A^ns^; H^ns^) (*p* < 0.001). Although the non sporulating variants produced less toxin, toxin production was still observed, suggesting the non sporulating variants are still toxigenic (Fig. 6). No significant difference in maximum toxin production was noted between A^ns^ and H^ns^ *(p* > 0.05). However, H^s^ produced significantly more toxin than A^s^ (*p* < 0.001).

### Motility assay

**Figure 7:**
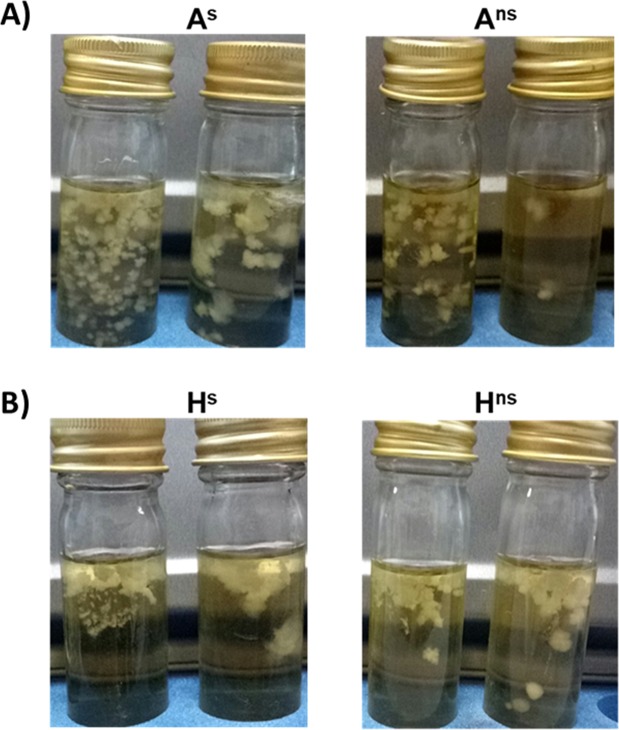
Motility of each isolate in BHI 0.175% agar for 24 h at 37 °C under anaerobic conditions. (**A**) Animal isolates: A^s^ and A^ns^, (**B**) Human isolates: H^s^ and H^ns^.

R078 is typically described as non-motile^24^ however motility was assessed to document if the secondary phenotype displayed any motility. Flocculent growth was observed from both phenotypes for both human and animal isolates in BHI (0.175%) agar (Fig. 7). This growth pattern indicates a non-motile organism as previously reported for R078^24^.

### Antimicrobial susceptibility to vancomycin and metronidazole

**Figure 8:**
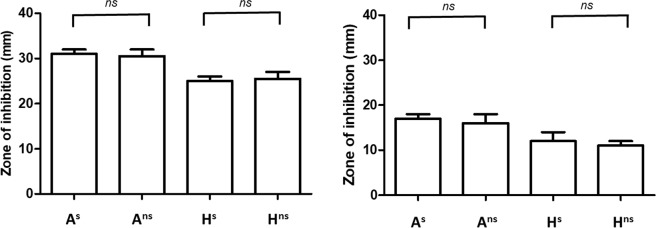
Susceptibility to metronidazole and vancomycin measured through zone of inhibition (mm). Isolates were grown anaerobically at 37 °C on FAABL for 24 h in the presence of antimicrobial discs containing 5 μg of (**A**) Metronidazole, (**B**) Vancomycin.

The susceptibility of each phenotypic variant to vancomycin and metronidazole was investigated as they are the primary antibiotics employed to treat CDI^25^. No significant difference was noted between A^s^ and A^ns^, and H^s^ and H^ns^, indicating each phenotype displayed similar susceptibility to both metronidazole and vancomycin. Overall, each isolate displayed more susceptibility to metronidazole than vancomycin, giving a larger zone of inhibition (p < 0.05) (Fig. 8).

Susceptibility to metronidazole was further assessed using MIC test strips. These confirmed the animal isolates were more susceptible to metronidazole with MIC of 0.5 μg compared to the human isolates with MIC of 0.75 μg. No difference in susceptibility to metronidazole between the sporulating and non-sporulating phenotypes was observed for either the human or animal derived isolates (p > 0.05).

### Illumina whole genome sequencing and analysis of *spo0A*

Purified DNA extracts of A^s^, A^ns^, H^s^ and H^ns^ were subject to Illumina sequencing, along with reference R078 isolate, M120. The aim was to assess any genetic differences between the isolates which may explain the emergence of the non-sporulating secondary phenotype. The sequencing statistics for each isolate showed a low number of contigs for a large genome, indicating good coverage and reasonably complete draft genomes (Supplementary Table S1).

**Figure 9:**
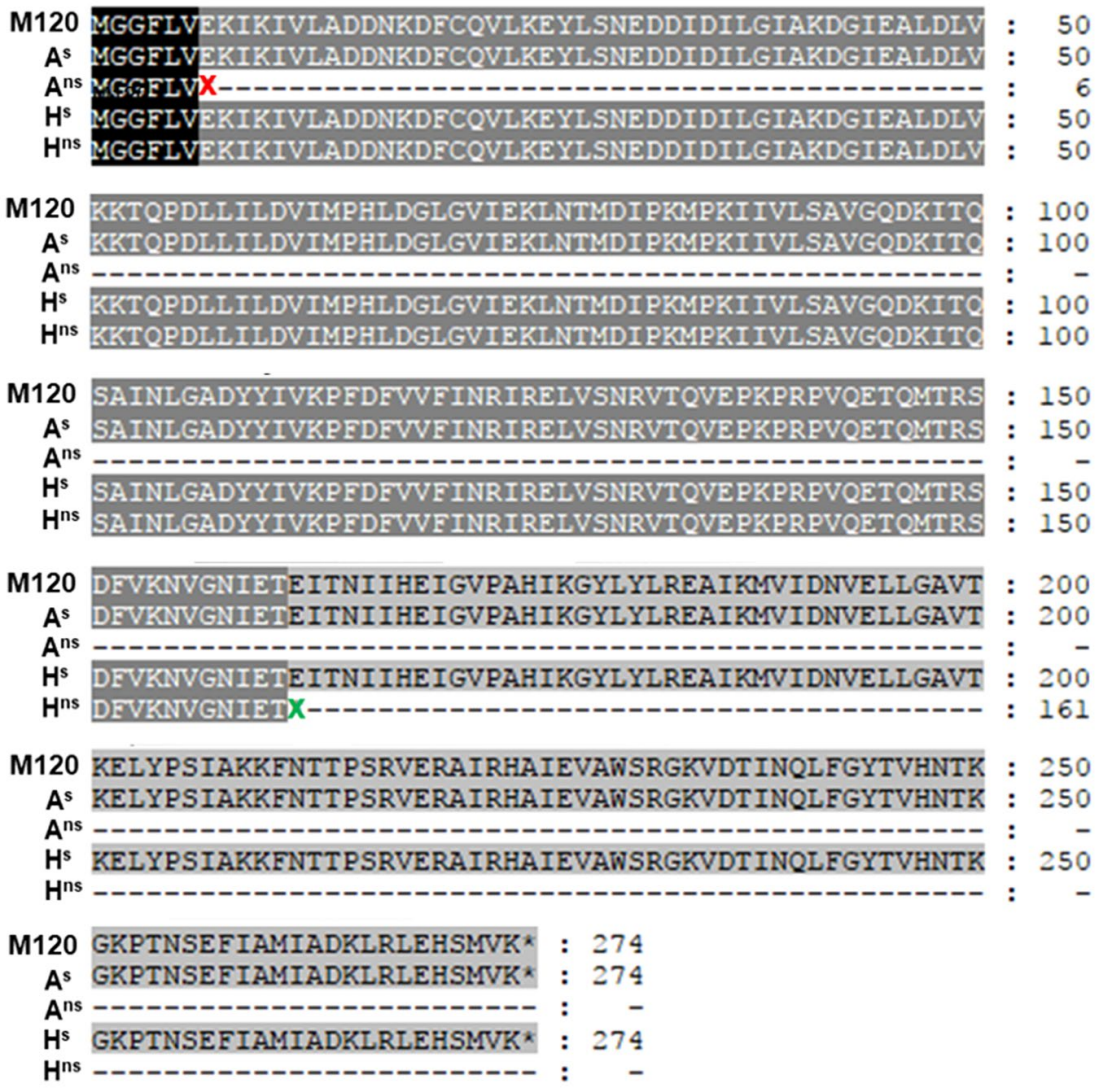
Alignment of sequences of *spo0A* for each isolate. **M120 –** reference 078, **A**^**s**^**-** animal isolate phenotype 1, **A**^**ns**^ **-** animal isolate phenotype 2 (with stop codon at the 7^th^ codon- **x**), **H**^**s**^ **-** human isolate phenotype 1, **H**^**ns**^ **-** human isolate phenotype 2 with stop codon at the 162^nd^ codon - **x**).

To investigate the sporulation differences seen between the sporulating and non-sporulating R078 variants, *spo0A* sequences were extracted from each assembly and for the sporulating M120 reference genome, using the SEED viewer interface. The entire *spo0A* gene was found to be present in each assembly. However, mutations that introduce stop codons into both the animal (A^ns^) and human (H^ns^) variants were found (Fig. 9).

A non-synonymous mutation was present in the A^ns^ *spo0A* gene, at the 7^th^ codon converting a glutamic acid codon (GAA) into a stop codon (TAA) (Fig. 9). Two mutations were identified in the H^ns^ *spo0A* gene, both also introducing stop codons. The first of these occurred at the 162^nd^ codon, again converting a glutamic acid codon (GAA) to TAA and the second at the 227^th^, also introducing a TAA stop codon. In both non-sporulating strains these mutations would be expected to result in truncated gene products (Fig. 9). These were the only non-synonymous mutations found within the assemblies, this is consistent with the MLVA analysis confirming the non-sporulating strains were mutants derived from the sporulating parental strain in each case. Within each assembly, the *spo0A* genes lay within large contigs with good (over 30X) coverage. This strongly suggests that the mutations observed do not appear to be sequencing errors: Sanger sequencing of the whole gene in each strain was also completed. No mutations were seen in the sporulating isolates, A^s^ and H^s^, which in turn were identical to the M120 reference strain (Fig. 9).

Within the *spo0A* gene of A^ns^, a stop codon in the N-Spo0A at the 7^th^ amino acid would be expected to terminate translation after expression of a short peptide with the sequence MGGFLV (Fig. 9). Within the *spo0A* gene of H^ns^, the stop codon at the 162^nd^ amino acid would allow expression of the entire N-domain, according to a study by Lewis *et al*.^26^. However, the C-domain would be absent (Fig. 9). This domain is the transactivation domain and is responsible for DNA binding and transcription activation. This would potentially prevent phosphorylation of Spo0A and affect transcription of several hundred genes, including sporulation^27^.

## Discussion

This study demonstrated that an asporogenous *C*. *difficile* phenotype emerges after prolonged incubation of some R078 strains on FAABL, *in vitro*. As well as conventional single colony subculture, MLVA analysis confirmed the observed phenotypic differences were not due to separation of distinct strains from mixed cultures (Table 1). Further study of the variants derived from one human and one animal R078 strain found no differences in growth rate, motility or vancomycin/metronidazole susceptibility relative to the original “wild type” strain. Illumina sequencing allowed for genetic comparison of the different phenotypes. The spo0A gene in both non-sporulating variants were found to harbour premature stop codons (Fig. 9), most likely resulting in truncation of the predicted gene product (Fig. 9).

As previously mentioned, non-sporulating variants have been observed in other species. For example, *Bacillus anthracis*, and other *Bacillus spp*., have been found to produce a non-sporulating variant *in vitro*, where nutrients are abundant and therefore the selective pressure to produce spores is low^22,28–30^. Colonies produced by the asporogenous variant of *B*. *anthracis* are translucent and larger than their sporulating counter parts, as seen with the non-sporulating *C*. *difficile* isolates in this study (Fig. 1). Furthermore, various mutations were also seen occurring at different points within the *spo0A* gene of the non-sporulating *B*. *anthracis* variants^22^. The emergence of this non-sporulating phenotype in *B*. *anthracis* appears to be solely due to conditions which do not promote stress induced sporulation – for example nutrient depletion, which has been determined as the key factor inducing sporulation in *B*. *anthracis*^22,31^. Abundance of nutrients could also explain the emergence of the non-sporulating R078 variant in this study as the R078 isolates were only grown on FAABL which is a nutrient rich medium. However, it is as yet unclear what exactly triggers sporulation in *C*. *difficile*. Environmental conditions such as nutrient starvation, pH and oxygen exposure may have a role to play^20^. With *B*. *anthracis*, reversion to the sporulating phenotype has not been completed through stress induced mechanisms, however sporulation has been restored in the asporogenous isolates through phage transduction^32^.

The non-sporulating mutants isolated in this study produced significantly less toxin than their sporulating counterparts (Fig. 6). The master regulator associated with sporulation is the Spo0A regulon, which has not only been found to regulate sporulation, but also motility, biofilm formation and many metabolic pathways such as glucose fermentation^17,20,23,33^. Mutations in the *spo0A* gene have previously been found to have an adverse effect on toxin production within *C*. *difficile*^34^. Mackin *et al*. found this effect on toxin production to be strain specific, as the molecular mechanisms for expression of the *C*. *difficile* toxin genes differ across phylogenetically distinct strains^35^. Inactivation of Spo0A has been seen to negatively affect toxin gene expression in some ribotype 027 isolates^35^.

However, in contrast to the results observed in this study, Mackin *et al*. reported that *spo0A* inactivation in the R078 isolate used in their study produced similar amounts of toxin when compared to the sporulating counter parts^35^. In this study those isolates with a mutation in the *spo0A* gene produced significantly less toxin than their sporulating counterparts (Fig. 6).

The results observed in this study are similar to that seen by Underwood *et al*., who reported toxin production was approximately 10% less in *C*. *difficile* 630 *spo0A* knock out isolates compared to the wild type *C*. *difficile* 630 isolate^34^. Variation in toxin production has been noted across different ribotypes, with increased toxin production linked to increased virulence and disease. Ribotype 027 has been shown to display increased levels of toxin production^36^ in comparison to other ribotypes such as R106, R001 and R012. However these isolates displaying ‘decreased’ levels of toxin production still have the ability to cause serious disease^36^ so the relationship between quantity of toxin produced and virulence is not straightforward. Studies in mice have found that *spo0A* mutant derivatives still caused intestinal disease however they lack the ability to persist outside the host, ultimately preventing transmission^15^. Therefore sporulation has been highlighted as a key factor in transmission of *C*. *difficile* and recurrence of CDI^15^.

In this study, the lack of sporulation did not affect motility or biofilm formation (Fig. 5; Fig. 7). Motility and biofilm formation are important characteristics of many bacterial species for both the colonisation and evasion of hostile environments^37^, their roles in *C*. *difficile* infection have been widely studied. Motility is not a characteristic shared by all *C*. *difficile* strains with Baban *et al*. reporting *C*. *difficile* isolates displaying varying degrees of motility^24^. *C*. *difficile* R20291 (epidemic R027) and *C*. *difficile* 630 (R012) are motile, however members of the R078 are known to be non-motile^24^. Biofilm formation and sporulation have been found to be linked in other bacteria^15,23^. This relationship has been studied extensively in *Bacillus subtilis* where it has been shown that biofilm formation is regulated by Spo0A^38^: Absence of a functional spo0A gene may result in lack of biofilm formation^17^. Though it was hypothesised that the mutation identified in *spo0A* of the non sporulating isolates would also affect biofilm formation, this was not the case. No significant difference in biofilm formation was detected between the sporulating and non sporulating isolates (Fig. 5).

Toxin production is a key factor contributing to the disease caused by *C*. *difficile*, however infection requires successful colonisation of the gut. The processes of adherence, colonisation and sporulation therefore play a particularly important role in infection. Furthermore, a number of spore proteins have been identified as pro-inflammatory immunogenic agents, suggesting they may also contribute to the pathogenesis associated with CDI^39–41^. Further research into the mechanism which allows successful *C*. *difficile* colonisation of the host is important, particularly due to the increase in recurrent infections being reported in 15–35% of cases of CDI^16,17^.

This study does not suggest a regulatory effect where the non sporulating population has ‘switched off’ sporulation. The ability to sporulate in these variants appears to be lost, rendering the vegetative cells vulnerable to aerobic conditions, thereby preventing transmission. In biological terms the emergence of non-sporulating phenotypes is counter-intuitive for a strict anaerobe such as *C*. *difficile* that relies on transmission via spores. Previous studies have investigated increased sporulation as a factor that increases virulence^42^. This study focused on only four isolates from R078, further investigation into other R078 isolates, along with other ribotypes would be just. The emergence of this non sporulating variant may be due to phenotypic heterogeneity, where potential benefits arise through the ‘division of labour’^43^. This phenomenon suggests that individual cells within a colony can display different phenotypic traits which may lead to interactions benefiting the colony over all^43^. Whether a selective advantage acting at a population level could explain the emergence of non-sporulating *C*. *difficile* phenotypes is unclear. The robust spores produced by *C*. *difficile* are what allows this pathogen to survive and persist in such a wide range of environments^44,45^. No obvious advantage in producing the asporogenous phenotype has yet been identified however. Further investigations are needed to determine if non-sporulating populations arises *in vivo*, and what role this may play in *C*. *difficile* infection and host colonisation.

## Materials and Methods

### Isolation of phenotypic variants

Human *C*. *difficile* R078 isolates were obtained from archives at the Kelvin Laboratories, Royal Victoria Hospital, Belfast. Animal *C*. *difficile* R078 isolates were cultured from animal samples supplied by the Veterinary Science Division (VSD), Agri-Food Biosciences Institute (AFBI), Belfast. Human (n = 5) and animal (n = 5). Each isolate was subject to at least two single-colony sub-cultures, to confirm these cultures were axenic. R078 isolates were grown on fastidious anaerobe agar with horse blood (FAABL; Oxoid, Basingstoke, UK) for seven days under anaerobic conditions (N_2_/CO_2_/H_2_) at 37 °C. Two distinct colony morphologies were observed with all ten R078 isolates tested. These could be separated and sub-cultured independently on FAABL. One human isolate and one animal isolate were selected for further investigation. Isolates were categorised based on their observed sporulation patterns as follows: animal sporulating phenotype (**A**^**s**^), animal non-sporulating phenotype (**A**^**ns**^), human sporulating phenotype (**H**^**s**^) and human non-sporulating phenotype (**H**^**ns**^). Cells/spores, from each colony morphology were harvested from FAABL and suspended in 20 μl sterile phosphate-buffered saline (PBS), smeared uniformly over a microscope slide and heat fixed. The cells/spores were then subject to staining for 10 min, in the presence of malachite green solution (0.5% [w/v]: Sigma Aldrich, Irvine, UK), according to method of Schaeffer and Fulton^46^. The slides were washed with dH_2_O and counter stained with Safranin (2.5% [w/v]: Sigma Aldrich, Irvine, UK) for 30 s. The slides were washed, air dried and viewed using oil emersion at x 1000 on a Leica DMR microscope (Leica Microsystems Inc., Milton Keynes, UK). Spores were identified by observation of green staining, while vegetative cells are red/pink.

### Multi-locus variable number tandem repeat analysis (mlva)

To further confirm cultures were axenic, and that separate colony types were not due to the co-occurrence of two otherwise similar strains, MLVA was used to analyse A^s^, A^ns^, H^s^ and H^ns^ isolates. The MLVA method described by Fawley *et al*.^47^ was employed. In brief, each repeat locus was amplified in a singleplex PCR, with a final volume of 10 μl. Each reaction contained; 1x Promega Flexi PCR Buffer (Promega, Southampton, UK), Nuclease Free Water (Sigma Aldrich, Irvine, UK), Bovine Serum Albumin (0.2 mg/ml) (Sigma Aldrich, Irvine, UK), MgCl_2_ (3 mM) (Promega, Southampton, UK), forward primer (0.05 µM) and reverse primer (0.05 µM) (Integrated DNA Technologies, Leuven, Belgium). PCR primers are shown in Table 2.

**Table 2:** MLVA primers according to Fawley *et al*.^47^.

Primer ID	Sequence 5′–3′	Repeat motif	Location
A6_F	FAM-TTAATTGAGGGAGAATGTTAAA	AAGAGC	755721
A6_R	AAATACTTTTCCCACTTTCATAA		
B7_F	ATTO550-CTTAATACTAAACTAACTCTAACCAGTAA	ATCTTCT	3688632
B7_R	TTATATTTTATGGGCATGTTAAA		
C6_F	FAM-GTTTAGAATCTACAGCATTATTTGA	TATTGC	3239736
C6_R	ATTGGAATTGAATGTAACAAAA		
E7_F	ATTO550-TGGAGCTATGGAAATTGATAA	ATAGATT	167124
E7_R	CAAATACATCTTGCATTAATTCTT		
F3_F	ATTO565-TTTTTGAAACTGAACCAACATA	TTA	1954915
F3_R	ACAAAAGACTGTGCAAATATACTAA		
G8_F	ATTO565-TGTATGAAGCAAGCTTTTTATT	TAAAAGAG	664660
G8_R	ACCAAAAATTTCTAACCCAAC		
H9_F	HEX-GTTTTGAGGAAACAAACCTATC	TCTTCTTCC	4116072
H9_R	GATGAGGAAATAGAAGAGTTCAA		

Thermal cycling conditions were as follows: 95 °C for 10 min, followed by 34 cycles of 95 °C for 30 s, 51 °C for 30 s and 72 °C for 30 s, followed by 72 °C for 5 min.

Post amplification, PCR products were diluted (1:10) with nuclease free water (NFW) (Sigma Aldrich, Irvine, UK). 1 μl of diluted PCR product was added to 9 μl Hi-Di formamide containing GeneScan 600 LIZ Size Standard (Applied Biosystems, Warrington, UK). Diluted samples were separated using capillary electrophoresis on a 3730XL Genetic Analyzer using POP-7 polymer and a 36 cm capillary array (Applied Biosystems, CA, USA). Fragment size data were analysed using GeneMarker software (SoftGenetics, LLC, USA) to assign MLVA profiles.

### Assessing sporulation in R078 colony variants

*C*. *difficile* R078 isolates designated as A^s^, A^ns^, H^s^ and H^ns^ were sub-cultured on FAABL under anaerobic conditions at 37 °C for 72 h. Colonies were harvested and cells immediately subjected to alcohol shock in 70% (v/v) ethanol for 1 h at room temperature^48^ to kill vegetative cells. Subsequently a 10-fold serial dilution (undiluted to 10^−3^) was carried out in sterile PBS (Sigma Aldrich, Irvine, UK) for each isolate. Aliquots of each dilution (100 μl) were plated onto Brazier’s agar (Oxoid, Basingstoke, UK) in triplicate, and incubated anaerobically at 37 °C for 48 h. A control experiment was carried out (working in the anaerobic chamber) where the cells were suspended in pre-reduced sterile distilled H_2_O for 1 h, as opposed to 70% [v/v] ethanol. An identical 10-fold serial dilution was carried out before plating onto Brazier’s agar, in triplicate. Visible colonies were enumerated after incubation to estimate Total Viable Counts (TVC) for each condition^49^.

### Growth rate analysis

Brain Heart Infusion broth (BHI) (Oxoid, Basingstoke, UK) was inoculated with a single colony of A^s^, A^ns^, H^s^ and H^ns^ and grown anaerobically for 24 h to an OD_600_ of 1.0, measured using an Eppendorf Biophotometer (Eppendorf, Stevenage, UK). Growth assays were carried out on technical replicate cultures (n = 12), grown in 96-well microtitre plates. BHI broth (180 μl per well) was aliquoted into sterile plates and pre-reduced in an anaerobic cabinet for 5 h prior to inoculation with 20 μl of a log phase broth cultures of each isolate. Uninoculated BHI-only controls (n = 12) were included in each plate. Plates were sealed in the anaerobic chamber and incubated at 37 °C using a FLUOstar Omega Microplate reader (BMG Labtech, Aylesbury, UK). Readings were taken every hour, with shaking for 20 s prior to OD_600_ measurements, for 24 h.

### Crystal Violet biofilm assay

A^s^, A^ns^, H^s^ and H^ns^ isolates were grown anaerobically on FAABL agar for 72 h. 5 ml BHI was pre-reduced for 5 h prior to inoculation with a single colony of each phenotype. Cultures were incubated anaerobically for 24 h at 37 °C.

Biofilm assays were carried out according to the method of Dawson *et al*.^23^. In brief, after 24 h growth, 20 μl of each strain was inoculated into 180 μl of pre-reduced BHI broth (1/10 dilution) with replication (n = 12) in microtitre plates. BHI-only controls were included for every well containing *C*. *difficile* (n = 12). Plates were sealed in the anaerobic chamber and incubated without shaking at 37 °C, for six days.

After incubation, media was aspirated and each well washed once with sterile PBS. 200 μl of 1% (v/v) Crystal Violet (Sigma Aldrich, UK) was added to each well and plates were incubated for 30 min at room temperature. After staining, wells were washed three times with sterile PBS. 200 μl of methanol (Sigma Aldrich, Irvine, UK) was added to each well to solubilise the remaining Crystal Violet, which will be representative of biofilm formation. After incubation at room temperature for 15 min, OD_595_ measurements were taken using a FLUOstar Omega Microplate reader (BMG Labtech, Aylesbury, UK).

### Toxin production

In order to confirm the ability to produce toxin was not affected by loss of sporulation, A^s^, A^ns^, H^s^ and H^ns^ isolates were grown anaerobically at 37 °C on FAABL for 72 h. 5 ml BHI broth was pre-reduced for 5 h prior to inoculation with a single colony of each isolate. Cultures were incubated anaerobically at 37 °C. Sampling began 14.5 h after inoculation, with samples taken every 3 h thereafter, for a total of 24 h, in triplicate. Presence of toxin in the cultures was evaluated at each time point using the Premier Toxins A&B kit (Meridian Bioscience, London, UK) in accordance with the manufacturer’s instructions, where optical density can be used to determine level of toxin present.

This assay is used to detect the toxins produced by toxigenic *C*. *difficile*, toxins A and B, using toxin specific antibodies. As per manufacturers instruction, 50 µl of broth culture was added to the antibody coated well followed by horseradish peroxidase (HRP)-conjugated anti-toxin A and B antibodies are added to wells. The presence of either toxin is detected the HRP-conjugated antibodies will bind to the toxins, which will remain in wells after washing. A chromagen is then added to the wells which will bind to HRP-conjugated anti-toxin and a blue colour will form. After 10 min, the reaction is stopped using 1 M Phosphoric acid. This colour intensity can be quantified using using a BMG FLUOstar Optima Fluorescence spectrophotometer (BMG Labtech Ltd, Aylesbury, UK) at OD 600 nm allowing for a positive or negative results to be recorded. Positive toxin production was indicated at OD_600_ ≥ 0.150.

### Motility assay

Motility was assessed using the qualitative assay of Stabler *et al*.^50^ with modification. A^s^, A^ns^, H^s^ and H^ns^ isolates were grown anaerobically on FAABL for 48 h. Working in the anaerobic chamber, triplicate colonies of each isolate were removed and resuspended in 50 µl sterile PBS (Sigma Aldrich, Irvine, UK) and a ten-fold serial dilution (undiluted to 10^−4^) was carried out. 10 ml of pre-reduced BHI containing 0.175% (w/v) agar was inoculated with each dilution, by carefully dropping 10 µl onto the surface, with minimum disturbance of the medium. Broths were incubated without shaking for 24 h under anaerobic conditions before being removed and photographed.

### Antibiotic susceptibility assay

A^s^, A^ns^, H^s^ and H^ns^ isolates were grown anaerobically at 37 °C on FAABL for 24 h. Cells were harvested and re-suspended in sterile PBS to an OD_600_ of 0.6. 200 μl of cell suspension was spread evenly onto FAABL (n = 3) and antibiotic test discs of metronidazole (5 μg) and vancomycin (5 μg) (Oxoid, Basingstoke, UK) were placed onto each plate. Plates were incubated anaerobically at 37 °C for 24 h. After incubation the zone of inhibition for each antibiotic was measured.

The Minimum Inhibitory Concentration (MIC) of metronidazole was also estimated using MIC test strips (Liofilchem, Teramo, Italy). A^s^, A^ns^, H^s^ and H^ns^ isolates were plated onto FAABL as previously described. The MIC test strip was placed on the plate and incubated anaerobically at 37 °C for 24 h. MIC was determined by reading the concentration of the MIC test strip where the zone of inhibition began, according to the manufacturer’s instructions.

### Statistical analysis

All statistical analyses were carried out using GraphPad Prism 5. One-Way Analysis of Variance (ANOVA) was employed to determine significant differences in sporulation, growth rate, antibiotic susceptibility, toxin production and biofilm formation within each of the corresponding sporulating and non-sporulating isolates. Statistical significance was determined at *p* < 0.05.

### Illumina whole genome sequencing

A^s^, A^ns^, H^s^ and H^ns^ were cultured overnight in BHI broth, along with reference isolate, M120. Broth cultures were centrifuged at 20,000 × g for 5 min and the cell pellets resuspended in 200 µl of MagNa Pure Bacteria lysis buffer (Roche Diagnostics, UK). DNA was extracted using the DNA/Viral Kit on the MagNA Pure Compact System (Roche Diagnostics, UK) in accordance with the manufacturer’s instructions. The purified DNA was eluted into a final volume of 100 µl, quantified using a Quantus fluorometer (Promega, UK) and concentrations normalised to 30 ng/µl. DNA samples were submitted for Illumina sequencing to the Microbes NG (University of Birmingham, UK) service. Samples were processed by MicrobesNG as follows: sequencing libraries were prepared using the Nextera DNA Sample Preparation Kit (Illumina, USA). Paired-end sequencing of the resulting libraries (2 × 250 bp) was carried out on the Illumina MiSeq™ platform (Illumina, USA).

Initial bioinformatics analysis was carried out by MicrobesNG. In brief, Kraken, a taxonomic sequence classification system, was used to identify the closest available reference genome – *C*. *difficile* 630. Sequence reads were mapped onto the reference genome using BWA-MEM (http://bio-bwa.sourceforge.net). De novo assembly was performed using SPades (http://bioinf.spbau.ru/spades) with mapping onto reference genome using BWA-MEM to assess quality.

Assemblies were analysed further using the RAST server^51^ (http://rast.nmpdr.org) to predict open reading frames (ORFs), followed by annotation using the SEED database^52^. Annotated assemblies were examined using the SEED Viewer.

## Supplementary information

Supplementary Dataset 1
