# Supplementary material for: Emergence of a non-sporulating secondary phenotype in Clostridium (Clostridioides) difficile ribotype 078 isolated from humans and animals
Source: Sci Rep. 2019 Sep 23;9:13722. doi: 10.1038/s41598-019-50285-y (PMC6757067; doi:10.1038/s41598-019-50285-y)
Supplement: Supplementary file 1 — Supplementary Dataset 1 [file 41598_2019_50285_MOESM1_ESM.pdf]

Emergence of a non-sporulating secondary phenotype in *Clostridium (Clostridioides) difficile* ribotype 078 isolated from humans and animals

Connor MC[1]*, McGrath JW[1], McMullan G[1], Marks N[1], Guelbenzu M[2] and Fairley DJ[3]

[1.] School of Biological Sciences and the Institute for Global Food Security, Medical Biology Centre, Queens University Belfast, UK; M.Connor@qub.ac.uk

[2.] Veterinary Science Division, Agri-Food Biosciences Institute, Belfast, UK (Current affiliation: Animal Health Ireland, Carrick on Shannon, County Leitrim, ROI).

[3.] Department of Microbiology, Belfast Health & Social Care Trust, Belfast, UK.

Supplementary Table S1: Sequencing statistics; the assembly metrics in the table below were calculated using QUAST by Microbes NG (University of Birmingham, UK)

**Statistics**	**H^{s}**	**H^{ns}**	**A^{s}**	**A^{ns}**
# contigs	48	89	53	51
Largest contig	467024	236341	466403	847071
Total length	3891245	3882917	3873841	3870230
N50	164925	101061	202407	188847
Mismatches (per 100kbp)	0	0	0	0
